# Distinct Regions of Right Temporo-Parietal Junction Are Selective for Theory of Mind and Exogenous Attention

**DOI:** 10.1371/journal.pone.0004869

**Published:** 2009-03-17

**Authors:** Jonathan Scholz, Christina Triantafyllou, Susan Whitfield-Gabrieli, Emery N. Brown, Rebecca Saxe

**Affiliations:** 1 School of Computer Science, Georgia Institute of Technology, Atlanta, Georgia, United States of America; 2 Department of Brain and Cognitive Sciences, Massachusetts Institute of Technology, Cambridge, Massachusetts, United States of America; Victoria University of Wellington, New Zealand

## Abstract

In functional magnetic resonance imaging (fMRI) studies, a cortical region in the right temporo-parietal junction (RTPJ) is recruited when participants read stories about people's thoughts (‘Theory of Mind’). Both fMRI and lesion studies suggest that a region near the RTPJ is associated with attentional reorienting in response to an unexpected stimulus. Do Theory of Mind and attentional reorienting recruit a single population of neurons, or are there two neighboring but distinct neural populations in the RTPJ? One recent study compared these activations, and found evidence consistent with a single common region. However, the apparent overlap may have been due to the low resolution of the previous technique. We tested this hypothesis using a high-resolution protocol, within-subjects analyses, and more powerful statistical methods. Strict conjunction analyses revealed that the area of overlap was small and on the periphery of each activation. In addition, a bootstrap analysis identified a reliable 6–10 mm spatial displacement between the peak activations of the two tasks; the same magnitude and direction of displacement was observed in within-subjects comparisons. In all, these results suggest that there are neighboring but distinct regions within the RTPJ implicated in Theory of Mind and orienting attention.

## Introduction

In fMRI studies, a cortical region in the right temporo-parietal junction (RTPJ) is recruited when participants read stories about people's thoughts, relative to controls for logical and attentional demands [1], [2]. The blood oxygen level dependent (BOLD) response in this region is significantly higher when participants read about false beliefs than during closely-matched stories about false maps or signs [3], and is specific to thinking about thoughts relative to other social information [4]–[6].

Both fMRI and lesion studies also suggest that a region near the RTPJ is involved in attentional reorienting. In a Posner spatial-cueing paradigm [7], activity in the RTPJ is increased on “invalidly-cued” trials, when a target appears in an unexpected location [8]. Similar activation has been observed during detection of centrally presented low-frequency targets [9], suggesting that the function of this region is in exogenously-cued redeployment of attention. Damage to RTPJ is the most common cause of left hemifield spatial neglect [10], [11].

One obvious question is: do these findings reflect recruitment of a single neural region, common to both Theory of Mind and exogenous attention, or are there neighboring but distinct regions within the RTPJ that account for these results? The discovery that these two tasks share a common mechanism would be surprising and informative, suggesting that the two tasks rely on common psychological component process.

Caution is clearly necessary before we accept an observation that two different tasks activate the “same” brain region in imaging studies as evidence that common psychological mechanisms are engaged. In standard fMRI methods, the large voxel-size, along with the combined effects of partial-voluming, pooling due to shared vasculature [12], imperfect alignment during normalization and group averaging, and spatial smoothing all conspire to bias fMRI analyses towards findings of spurious overlap [13].

Mitchell [14] recently conducted the first direct comparison of the Theory of Mind and exogenous attention tasks in the same individuals. He reported overlap between the regions of RTPJ recruited by the two tasks. For the reasons described above, these results are potentially important but hard to interpret. We therefore investigated whether there is real overlap between regions recruited for Theory of mind and attentional reorienting, and/or whether there is a reliable spatial separation between the regions activated by the two tasks, using higher field strength (3T versus 1.5T) and higher resolution (1.6×1.6×2.4 mm voxels, versus 3.75×3.75×6 mm) than the previous study. To overcome the limitations of the weak attention effect, we use a non-parametric bootstrap to estimate confidence intervals for the relative locations of the peaks in group data. The bootstrap is a computationally intensive method for assessing the uncertainty in a statistical measure [15].

## Methods

Twenty-one naive adults (ten women, eleven men) participated in the study for payment. Participants gave written consent, as approved by the Committee on the Use of Humans as Experimental Subjects at the Massachusetts Institute of Technology. All subjects were right-handed, native English speakers, and had normal or corrected-to-normal vision. Subjects were scanned using 12-channel head coil, in a 3T Siemens MAGNETOM Trio, a TIM system (Siemens Medical Solutions, Erlangen, Germany) in the Athinoula A. Martinos Imaging Center at the McGovern Institute for Brain Research at MIT. Functional BOLD images were collected using a single shot gradient echo echo-planar imaging (EPI) sequence with TR = 2s, TE = 30 ms, flip angle = 90°. Twenty-one 2-mm thick near-axial slices were acquired with 20% interslice gap and in-plane resolution of 1.6×1.6 mm, providing coverage over superior temporal and parietal cortices.

**Figure 1:**
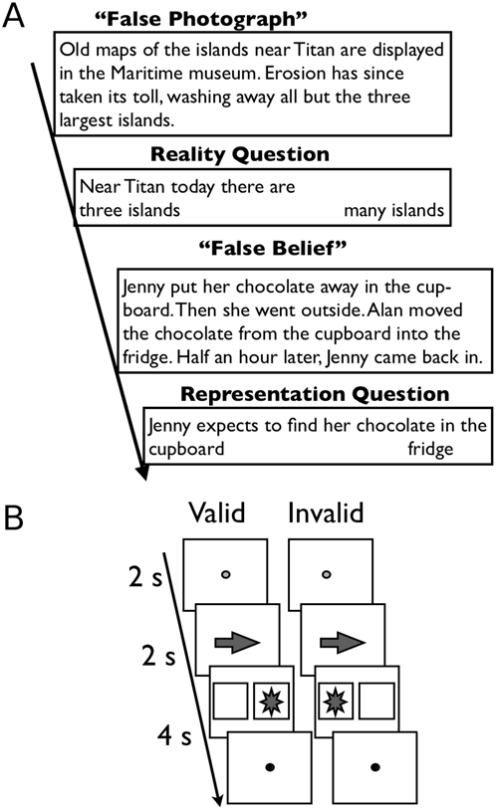
Task structure for the two tasks. (A) Sample stimuli for the Theory of Mind task. The key contrast compares the brain response while participants read ‘Belief’ vs ‘Photo’ stories. (B) Four trial types in the attentional reorienting task. The key contrast compared the brain response while participants detect the target on Invalid, vs Valid, trials.

Runs of two experiments were interleaved in a single scan session for each participant. In the Theory of Mind experiment, subjects read 24 short vignettes about the formation of a representation (12 about beliefs [‘Belief’ trials], 12 about physical representations like a photo, drawing, or map [‘Photo’ trials]) that did not correspond to a reality [2,Figure 1a]. Stories were on average 32 words long, and were presented for 10 seconds. Subjects then answered a fill-in-the-blank question either about the representation or about reality (4 seconds). Stories from the two conditions alternated (order counterbalanced across subjects and runs), with 12s rest interleaved between each story. Each run lasted 2 min and 48 seconds (six stories); each subject participated in four runs of this experiment.

The spatial attention experiment followed the procedure described by Corbetta [8], adapted from the Posner cueing task [7]. Before each trial, the subject viewed a central fixation dot flanked on either side by empty square boxes. At the start of a trial the fixation dot turned green (Figure 1b), and was followed by a cue arrow pointing to one side (left 50%, right 50%). The arrow was followed in 44% of the trials by the appearance of the target (a large asterisk) at the cued location (a “valid” trial), in another 16% of the trials at the opposite location (an “invalid” trial), and in 20% of trials, no target appeared (a “noise” trial). On the remaining 20% of trials, after the arrow appeared the central fixation turned immediately back to red (“cue” trial). Trials were separated by a fixation interval of 9, 10 or 12 seconds (randomly distributed; mean fixation duration 10 seconds). Trials from the four conditions (valid, invalid, cue, noise) were randomly intermixed. Each run was comprised of 25 trials, and each subject participated in 4 to 6 runs of the experiment. Subjects were provided an MR-safe response box and instructed to respond with a single button press as quickly as possible when a target appeared in either box (effectively, a go/no-go task). This method isolates the effect of “invalid” trials to visuo-spatial attention; there is no response conflict once the target has been detected.

Stimuli were presented onto a screen via Matlab 5.0 with an Apple G4 powerbook on a dark background. Reaction time measures were obtained during both experiments with the MR-safe button box. Behavioral data for three subjects in the Theory of Mind task were lost due to technical error.

fMRI data were analyzed with SPM2 (Wellcome Department of Cognitive Neurology, London, UK). The images were motion corrected, normalized to a functional template, and smoothed with a Gaussian filter (FWHM = 2.5 mm) prior to analysis; during normalizing, the functional data were resliced to 2×2×2 mm. Data were then high-pass filtered and modeled using a boxcar regressor to estimate the hemodynamic responses for each condition (Belief, Photo; Invalid, Valid, Cue, Noise). Contrasts from each task (Belief-Photo, Invalid-Valid) were calculated for each subject and then combined for group random effects analyses. Surface reconstructions, for the figures, were constructed for the group average results based on a standard single subject anatomical.

An analysis of overlap between activations was conducted on group random effects data using in-house software. Overlap was calculated as a function of threshold on the voxel-wise T statistic for the two contrasts. For the purposes of the overlap analyses, an anatomical ROI was defined as a cube around the peaks from the two group averages: [X 40∶66; Y −73∶−41; Z 20∶52]. Active voxels were defined as any voxel passing the threshold in either task that fell inside the anatomical ROI. Overlap was measured as a strict conjunction: each voxel counted as ‘overlap’ only if the contrast exceeded the T-threshold independently for both tasks. Percentage overlap was defined as the percentage of active voxels that were counted in the conjunction. For technical reasons, in the bootstrap and individual subjects overlap analyses there was no extent threshold applied, whereas in the group analyses used an extent threshold of k>10; as a result the bootstrap analysis was more likely to find overlapping voxels, and therefore provides an upper bound on the true percentage overlap.

The spatial relationship between activations was assessed with a repeated-sample random effects analysis using a non-parametric bootstrap. In this procedure the data were randomly sampled, with replacement, to create 150 bootstrap samples (each with n = 21). Random effects analyses were generated with each of these samples for each task contrast. Peak voxel coordinates were selected automatically for each sample as the voxel with the maximum T-statistic within an 18 mm radius of the anatomically-defined centre of the RTPJ.

## Results

### Behavioral Results

Reaction times during the attention task were faster on valid (mean 408 ms) than invalid trials (445 ms) using a paired-samples t-test, confirming that subjects were influenced by the spatial cue (t(20) = 3.8,p<0.001). Participants' responses to Belief trials (2.64 s) were faster to Photo trials (2.80 s) during the Theory of Mind task (t(17) = 2.7,p = 0.015).

### fMRI Results

**Figure 2:**
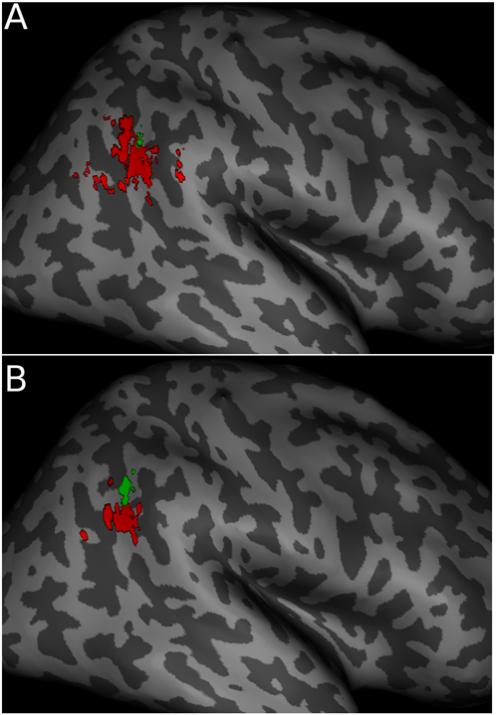
Group Activations. (A) Group activations for Belief – Photo (Red) and Invalid – Valid (Green), both p<0.001 uncorrected, k>5. (B) Group activations with approximately matching numbers of voxels: Belief – Photo (Red, 82 voxels, p<0.0001, k>10) and Invalid – Valid (Green, 72 voxels, p<0.005, k>10). (C) Interaction of the two contrasts, ((Belief-Photo)-(Invalid-Valid)), p<0.01, k>10. All activations were superimposed on the inflated T1 canonical brain in SPM using the SurfRend toolbox.

Consistent with previous results, random effects group analyses revealed regions near the right temporo-parietal junction recruited during the Theory of Mind task (Belief>Photo, peak voxel: 60 −56 32 (MNI), cluster size: 320 voxels (2.56 cm^3^) at p<0.001, peak T = 7.5) and the spatial attention task (Invalid>Valid, peak voxel: 58 −62 42, cluster size: 16 voxels (0.13 cm^3^) at p<0.001, peak T = 5.9, Figure 2a). Each of these peaks was somewhat higher than the average peaks reported by Mitchell [14] and Decety and Lamm [16], who for example report peaks for Attention at [52 −50 28], and for Theory of Mind at [52 −52 18]. This difference within the range of inter-subject variability in anatomy [9], and illustrates the need for within subject comparisons across tasks to reveal their true functional relationships.

In individual subjects, a cluster of k>10 contiguous voxels, at a threshold of p<0.001, was identified near the RTPJ in 18/21 individual subjects for Belief>Photo, and 12/21 individuals for Invalid>Valid. Eleven individuals had clusters in both contrasts; in those individuals, the average cluster size for Belief>Photo was 268 voxels (2.15 cm^3^), and for Invalid>Valid was 52 voxels (0.42 cm^3^).

The current design allowed us to ask four questions about the relationship between brain regions recruited during the Theory of Mind and attention tasks. First, can we replicate the prior finding that the RTPJ region implicated in each task does significantly differentiate, on average, the critical trials of the other task? Second, is there a region significantly recruited for the conjunction of both tasks (i.e. is there any real overlap)? Third, within the regions recruited by each task, are common or distinct sub-populations of neurons driving the responses to the two tasks? Finally, is there a reliable spatial separation between the peak activations for the two tasks? To answer these questions, we analyzed the results in the group average results, using a bootstrap to estimate confidence intervals, and separately in the eleven individual subjects in whom both contrasts yielded detectable activations.

#### ROI analyses – Group Results

We defined functional regions of interest for each task in group data and then examined the functional response to the other task in these regions. The ROI defined by the Theory of Mind task did differentiate between invalid and valid trials of the attention task (t(20) = 4.9,p<0.0001), and the ROI defined by the attention task differentiated between belief and photo trials of the Theory of Mind task (t(20) = 3.8,p<0.001), consistent with previous results [14].

#### ROI analyses – Individual Subjects

**Figure 3:**
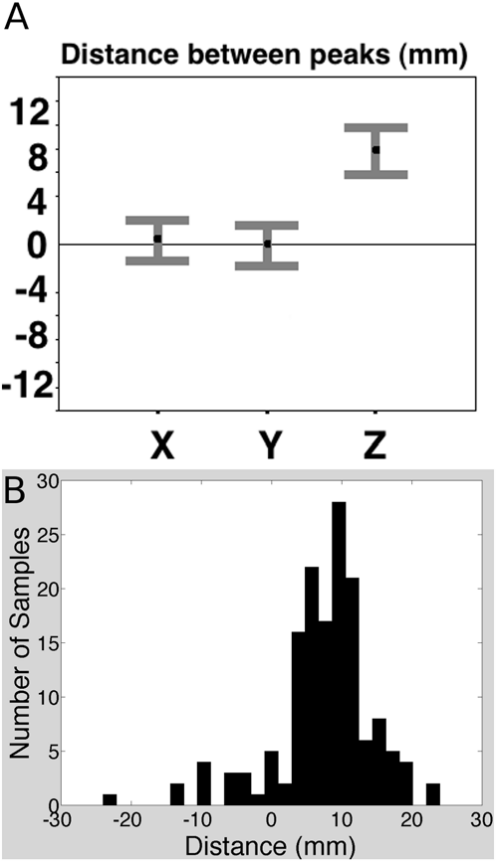
Spatial Separation. (A) Distance between the peaks estimated by the bootstrap: attention region – Theory of Mind region. Bars show 99.9% confidence intervals (3.36 standard errors of the bootstrap mean). There was no reliable difference in the X (medial to lateral) or Y (anterior to posterior) axes, but the attention region was reliably 6–10 mm superior to the theory of mind region. (B) Histogram of the observed distance, on the Z dimension, between the Theory of Mind and attention regions, in the 150 non-parametric bootstrap samples. Positive values indicate that the attention peak was superior to the Theory of Mind peak.

We also conducted an ROI analysis in the eleven individual subjects who showed an exogenous attention effect. In these data we observed the same pattern that we found in the group data, but with smaller effects. The ROI defined by the Theory of Mind task differentiated between the invalid and valid trials of the attention task (t(10) = 3.3,p<0.01), and the ROI defined by the attention task differentiated belief and photo trials of the Theory of Mind task (t(10) = 3.9,p<0.01) (Figure 3).

#### Overlap – Group Results

In order to determine the amount of overlap between the group average activations, we calculated the number of voxels in the group whole brain contrast recruited for both tasks as a function of statistical threshold. For T>3.5, p<0.001, there were 7 voxels in the conjunction, accounting for 2% of active voxels. This percentage decreased with increasingly strict T-thresholds as long as the number of overlapping voxels was non-zero. We also calculated the overlap between these two activations after correcting for the difference in size between the two regions. The Belief versus Photo contrast was set to a threshold of p<0.0001, k>10, and the Invalid versus Valid was set to a threshold of p<0.005, k>10. These thresholds produced activations including 82 voxels (0.66 cm^3^) in the Theory of mind region, and 72 voxels (0.58 cm^3^) in the attention region. Using these criteria, 5% of active voxels passed both thresholds (Figure 2b).

#### Overlap – Bootstrap

In order to estimate confidence intervals for the percent overlap, we calculated the percent overlap between the two activations, at T>3.5, p<0.001, k>0, in each bootstrap sample. This analysis yielded a 99.9% confidence interval of 6–8% mean overlap.

#### Overlap – Individual Subjects

For each of the eleven individuals in whom we could identify a cluster in both contrasts at p<0.001, k>0, we calculated the percent of active voxels that were in the conjunction. The average percent overlap in these individual subjects was 3%; the range was from zero overlap to 10% overlap.

#### Cross-Voxel Correlations – Group Results

Peelen et al [17] recently introduced a cross-voxel correlation analysis, specifically designed to examine whether two functional responses within a single fMRI region arose from common or distinct neural sub-populations. In this analysis, the t-value for each contrast was extracted from each individual voxel within the region of interest. If the t-values from two contrasts were positively correlated across voxels, then the two contrasts were inferred to share common sub-populations of neurons within the region. In the size-matched ROIs defined by the Group Results, we found no evidence for a cross-voxel correlation between the t-values for the Belief-Photo and Invalid-Valid contrasts (Theory of Mind region, r^2^ = 0.03; Attention region, r^2^ = 0.01).

#### Spatial Separation – Bootstrap

**Figure 4:**
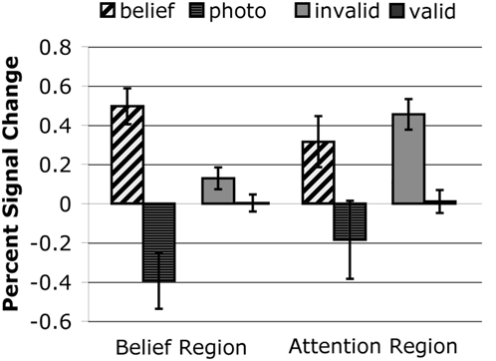
Percent signal change in individually defined regions of interest (n = 11). Bars represent standard error of the mean.

In the group random effects analyses, the voxel most significantly recruited by the belief task was 2 mm lateral, 6 mm anterior, and 10 mm inferior to the peak voxel in the attention task. The bootstrap revealed that only the spatial displacement in the Z (inferior-superior) dimension was reliable. There was no reliable difference between the location of the two peaks in the X (medial - lateral) or Y (anterior-posterior) dimensions (confidence intervals on the differences included 0). However, the peak for the attention task was reliably superior to the peak for the belief task (99.9% confidence interval of the mean for the z-coordinate of the attention task: 38–42 mm; of the belief task 31–33 mm; 99.9% confidence interval for the mean difference between the peaks within a sample: 6–10 mm, Figure 4).

#### Spatial Separation – Individual Subjects

In the 11 individual subjects in whom clusters could be identified for both contrasts, at p<0.001, the average peak of the cluster in Belief>Photo was at [57 −63 33]; in the same subjects, the average peak of Invalid>Valid was at [55 −60 41]. The peak of the attention cluster was on average 9 mm superior to the peak of the Theory of Mind cluster in the same individual (t(10) = 2.6, p = 0.03, paired-samples t-test).

## Discussion

The specific question at the center of this paper is whether one common neural substrate near the RTPJ is recruited both during the Theory of Mind and exogenous attention tasks. Ideally, any substantive claim for a common neural mechanism requires evidence of substantial overlap and no reliable spatial separation between the regions activated by the two tasks, in individual subjects' brains. These standards of evidence are hard to achieve in the current case, because the region recruited by attentional reorienting is smaller and less reliable in individual subjects that then region recruited during Theory of Mind tasks [2], [14]. At any statistical threshold, partial overlap might be evidence that the two tasks recruit the same region to different degrees, or that the two tasks recruit neighboring but distinct neural populations whose peripheries are partly overlapping. To overcome these limitations, we used three analysis strategies: a direct comparison of the two regions in the eleven individuals in whom both regions could be identified, a cross-voxel correlation analysis within each region of interest, and group analyses using a bootstrap to estimate confidence intervals. All three analyses converged to suggest that Theory of Mind and attentional reorienting recruit distinct cortical regions near the RTPJ.

Mitchell [14] recently reported that the activations associated with the two tasks, in group analyses, were substantially overlapping, and that ROIs defined by the Belief-Photo contrast significantly differentiated between Invalid and Valid trials of the attention task. We replicated this latter result in the ROIs, but we found much less evidence of overlap in direct tests. Specifically, cross-voxel correlation analyses suggested that distinct sub-populations within each region were driving the responses to the Belief-Photo, and Invalid-Valid, contrasts. The ROI average results may therefore reflect “bleed” of the functional response between two nearby regions, or neural populations. In an ROI analysis, the functional responses of all voxels in the region are averaged together, so relatively few voxels with an overlapping response may be sufficient to generate a significantly different average response. This is also consistent with the observation that the magnitude of the attention effect was small in the belief region, and vice versa (Figure 3), and with strict conjunction analyses suggesting that the overlap between the two regions is relatively small and at the periphery of the two activations (Figure 2).

Given these results, we suggest that the substantial overlap observed in the previous study may have been partly due to partial voluming effects in lower resolution data. Distinctions between nearby functional regions that are conflated in low resolution data can often be differentiated at higher resolution (e.g. [12], [18]). For example, response inhibition tasks yield bilateral activation in the anterior cingulate at low resolution (2×2×4 mm) but strongly right lateralized activation at high-resolution (1.5 mm isotropic, [19]. In the current study, the separation between the peaks of the two regions was estimated to be 6–10 mm, approximately two voxels at the resolution of the previous paper (3.75×3.75×6 mm, [14], making these regions nearly impossible to resolve. The higher resolution we used (1.6×1.6×2.4 mm) was probably a key factor making the difference between the current and previous conclusions in the overlap analyses.

One challenge for overlap analyses, though, is that the two functional contrasts may not be matched in power. In fact, it is unclear how to compare the power of the two experiments. Some considerations favor the attention effect. We measured the response to 16–24 invalid attention trials per individual, and only 12 belief trials per individual, allowing for a more accurate estimate of the amplitude of the response to invalid vs. valid trials; and our temporal model for the invalid cue (the onset of the target) was more precise than for the onset of belief representation, allowing for better prediction of the hemodynamic response in the attention task. On the other hand, the sentences implying a character's beliefs were presented for 10 seconds, whereas the target in the attention task was presented for less than a second. Since the latency, reliability, and duration of the two cognitive processes (detecting that a cue was invalid, constructing a belief representation) are both unknown, a precise estimate of the relative power of the two experiments is hard to derive. In subsequent analyses, we therefore analyzed the position rather than the extent of these activations. Because this approach utilized only peak coordinates it was relatively immune to differences in power across the experiments.

We used a non-parametric bootstrap procedure to estimate the relative spatial positions of the two regions of activation (Figure 3). The voxels showing the strongest response in the two tasks, in the group Random Effects analyses, were separated by 2×6×10 mm. However, traditional group analyses do not provide a way to estimate confidence intervals for this measurement. The bootstrap technique is particularly useful for estimating the distribution of a statistic in a situation like this one in which a measurement can be obtained from a group average but not from individuals. Bootstrapping is a simple, widely used, but computationally intensive method for estimating uncertainty in statistics of interest [20], [21]. Although quite common in the broader science community, to our knowledge this is the first time the bootstrap has been used to estimate confidence in the location of peak activations in fMRI data (but see [22]).

Non-parametric samples for the bootstrap are constructed by sampling randomly with replacement from the population; the average peak location (or other statistic) is then calculated for each task contrast. Since individuals from the original sample contribute differentially to each bootstrap sample (i.e. in any one bootstrap sample of size n, each of the original individuals is represented between zero and n times), the variability of the means across the bootstrap samples provide an estimate of the variability across individuals in the original sample. Efron and Tibshirani [15] report that 25 to 200 bootstrap samples may be required to accurately compute the bootstrap estimate of the standard error of a parameter like the peak locations in the current study; we used 150 samples. This technique proved very useful. We were able to estimate that while the observed separation between the peaks in the anterior-posterior and medial-lateral axes was not reliably greater than zero, there was a highly reliable segregation of the peaks of the two regions in the inferior-superior axis. The same direction and magnitude of separation was apparent in the eleven individual subjects in whom activated clusters could be detected in both contrasts.

The inferior-superior segregation of the two activations in the current data is also consistent with the results of previous studies. First, Mitchell [14] reported that the average peak of the Theory of Mind regions in three previous papers was [56 −54 19], and the average peak of the attention region in five previous papers was [55 −50 26]. The attention region was therefore on average 7 mm superior to the belief region, consistent with the distance estimated by our bootstrap. Second, Decety and Lamm [16] recently conducted a meta-analysis of seventy previously published group analyses of attention and Theory of Mind. Again, the authors reported that attention tasks produced an average peak activation 10 mm superior to the average peak of Theory of Mind tasks. Decety and Lamm [16] concluded that a spatial separation of 10 mm was consistent with a single underlying cortical region. However, the convergence of the meta-analysis with our current results is more consistent with a real dissociation between two distinct regions.

In sum, Theory of Mind and exogenous attention appear to recruit neighboring but distinct regions of cortex. These results are consistent with the (intuitive) idea that these tasks do not share a common cognitive component process - although it is of course still possible that these two regions are neighboring because they are functionally or ontogenetically related to one another.

Note that prior data already made it very unlikely that the results of either paradigm included a confound of the other. The attention task is does not covertly depend on Theory of Mind [14]. RTPJ recruitment has been observed for exogenous attention tasks that don't involve any “false cueing” manipulation [9]. Similarly, the results observed in Theory of Mind tasks are not confounded with shifts of exogenous attention. False photographs and false maps provide a well-matched control for false beliefs in terms of logical and inhibitory demands, as well as reading times and syntactic complexity, but do not recruit the RTPJ [2], [3], [23]. The response of the RTPJ for Theory of Mind tasks generalizes from verbal to pictorial stimuli [24], and from visual to aural presentation (Bedny et al, in preparation), each of which creates different attentional demands. The initial response of the RTPJ is specific in time to the moment when a belief is presented, but independent of both the truth-value and the emotional valence of the belief content [5], [25]; that is, the response is equally high for true and false beliefs, for negatively- or positively-valenced beliefs, and for beliefs shared or not shared by the participant. Finally, even when the stimuli and the subjects' responses are all physically identical, just changing the task instructions from an abstract rule to answering a question about a person's thoughts is sufficient to elicit enhanced recruitment of the RTPJ [6]. Overall, this profile cannot be explained away in terms of attentional shifts; and instead suggests a neural mechanism involved in thinking about thoughts and beliefs.

The question for the current paper was therefore not whether the tasks used in prior studies were confounded. Instead, evidence of a common neural region would have suggested the presence of a component process, not evident from intuitive task analyses, but shared by both tasks. In principle, this kind of evidence could be an important contribution of fMRI to cognitive science. However, the current results illustrate some of the challenges for establishing that two dissimilar tasks share a common neural substrate based on overlapping activations in fMRI data. The low resolution of typical fMRI data relative to the true functional resolution of cortex, along with the combined effects of partial voluming, pooling due to shared vasculature [12], distortions during normalization for group averaging, and spatial smoothing all conspire to bias fMRI analyses towards findings of spurious overlap. In the specific case under investigation here, the regions of RTPJ implicated in exogenous attention and Theory of Mind, our results suggest that the regions recruited for the two tasks are nearby but distinct, and consequently there is no need to posit a common psychological mechanism.[Table pone-0004869-t001]

**Table 1 pone-0004869-t001:** Direct comparison of the activations for Theory of Mind and attentional reorienting.

	Group Average	Bootstrap Estimate	Individual Subjects
**Percent Overlap, both p<0.001**	2%	6–8%	3%
**Distance between Peaks**	10 mm	6–10 mm	9 mm

Overlap and spatial separation between the Theory of Mind and attention regions, computed in the group average, using a non-parametric bootstrap to estimate confidence intervals, and in eleven individual subjects.
